# Requirements, Deployments, and Challenges of LoRa Technology: A Survey

**DOI:** 10.1155/2023/5183062

**Published:** 2023-01-09

**Authors:** Muhammad Ayoub Kamal, Muhammad Mansoor Alam, Aznida Abu Bakar Sajak, Mazliham Mohd Su'ud

**Affiliations:** ^1^Malaysian Institute of Information Technology (MIIT), Universiti Kuala Lumpur, Kuala Lumpur 50250, Malaysia; ^2^Institute of Business and Management, Karachi 75190, Pakistan; ^3^Riphah Institute of System Engineering (RISE), Faculty of Computing, Riphah International University, Islamabad 46000, Pakistan; ^4^Faculty of Computing and Informatics, Multimedia University, Cyberjaya, Malaysia; ^5^Faculty of Engineering and Information Technology, School of Computer Science, University of Technology Sydney, Australia; ^6^Malaysian France Institute (MFI), Universiti Kuala Lumpur, Kuala Lumpur 50250, Malaysia

## Abstract

LoRa is an ISM-band based LPWAN communication protocol. Despite their wide network penetration of approximately 20 kilometers or higher using lower than 14 decibels transmitting power, it has been extensively documented and used in academia and industry. Although LoRa connectivity defines a public platform and enables users to create independent low-power wireless connections while relying on external architecture, it has gained considerable interest from scholars and the market. The two fundamental components of this platform are LoRaWAN and LoRa PHY. The consumer LoRaWAN component of the technology describes the network model, connectivity procedures, ability to operate the frequency range, and the types of interlinked gadgets. In contrast, the LoRa PHY component is patentable and provides information on the modulation strategy which is being utilized and its attributes. There are now several LoRa platforms available. To create usable LoRa systems, there are presently several technical difficulties to be overcome, such as connection management, allocation of resources, consistent communications, and security. This study presents a thorough overview of LoRa networking, covering the technological difficulties in setting up LoRa infrastructures and current solutions. Several outstanding challenges of LoRa communication are presented depending on our thorough research of the available solutions. The research report aims to stimulate additional research toward enhancing the LoRa Network capacity and allowing more realistic installations.

## 1. Introduction

There are several uses for evolving wireless sensor networks (WSNs) in business, education, commuting, infrastructure, and protection [1]. Massive, widely dispersed sensing gadgets having data management capabilities, sensors, actuators, energy harvester devices, various forms of memory, radio signal-based transmitters, and receivers are all included in WSNs [2–4]. Such networked devices are placed across a sizable operational region to acquire data by detecting or monitoring the testbed. The central computer then receives the data for analysis [5]. It follows because while the IoT industry grows, it will undoubtedly draw wider and wider emphasis on itself. The Internet of Things (IoT) gadgets seek to provide services with long distance mobile gadget interconnection methods [6, 7]. Several emerging IoT upcoming developments are low—powered large area networking, which includes several standards and concepts including Sigfox, long range, narrowband fidelity, narrowband-IoT, and weightless (which combines several low-power wireless principles) [8]. Solutions for the IoT use gadgets with smart sensors to detect their surroundings, connect among various “things,” and execute wise judgments [9]. Wireless communication must offer reliable services, greater range, and good battery consumption to link IoT gadgets [10]. Within a given channel bandwidth, data throughput is exchanged for sensitivity in the LoRa unique spread spectrum encoding technique, which is a derivative of Chirp Spread Spectrum Coding [11]. LoRa comprises two layers: a MAC level algorithm and a physical layer using CSS modulation [12]. This technology can also broadcast in the ISM bands below 1 GHz [13]. The LoRa Alliance, a collection of the world's most powerful businesses in the communications sector [14], created the underlying technology (protocol), which includes, but is neither confined to SEMTEC and IBM, to highlight not many [15]. The CSS modulation that utilizes LoRa is proprietary [16]. As a result of this modulation method, LoRa is more resistant to interference and noise, making it more difficult to connect or recognize [17]. Narrowband signals are stretched over a larger band with this approach, making it less effective in spectrum usage while increasing connection cost and enhancing resistance to signal distortion, multipath fading, and Doppler effects [18]. To allow the scheme to change, LoRa employs six spreading factors ranging from SF7 to SF12 for its data rate and range. It also enables padding of the data packets delivered, making them more noise resistant. Higher spreading factors contrasted with increased delay [19]. It is important to note that, like many other LPWAN technologies, LoRa uses a “wait and listen” method to make it half duplex. The maximum message packet size for LoRa is 243 bytes when using the LoRaWAN protocol [6]. Because of their resilience to jamming, spread spectrum approaches have got much attention. The technology's capacity for communication is deemed if it has a higher information transfer capacity than is necessary using a spread spectrum. Furthermore, considering the features of spread spectrum modulation, we proposed the following criteria:

- A “spreading signal or coding signal” performs the “spreading” operation. The data being transferred has no bearing on this signal.
- The transmission bandwidth is far more than the least bandwidth necessary to convey data.
- Signal recovery is accomplished at the receiver using a replica of the coding sequence that was used to disseminate the supplied data to compare the stretched signal.

Overall performance gains by increasing the channel's bandwidth. LoRa supports data speeds of 300 bps to 37.5 kbps. The LoRa Alliance created the LoRaWAN MAC layer standard in 2015 [20]. Some technical details of LoRA are shown in Table 1.

LoRa systems have indeed been extensively employed for a wide range of industrial and learning systems. LoRa is an excellent choice for many IoT projects because of its versatility [22]. Smart girds [23], smart meters [24], smart street lights [25, 26], smart cities [27, 28], water quality [29], smart agriculture [30–32], temperature, and soil monitoring system [33–35] are examples of LoRA-based IoT applications.

- LoRA has a wider coverage zone that can be covered by a metropolitan area implementing the LoRa technology.
- LoRa's implementation can support a low-cost market since it uses an unlicensed frequency.
- It consumes very little power and does not require charging because the battery lasts around five years.

LoRa seems to be a modern tech on the market that operates in the nonlicensed spectrum below 1 GHz for long-range network communication. Within a given available spectrum, LoRa is a private spread spectrum modulation system, that is, a variant of CSS that trades data throughput for sensibility. Since its development in the 1940s, CSS has been employed in military uses due to its long transmission range and interfering resistance. The first low-cost, commercially viable implementation is LoRa. The benefit of long-range capability is where the word LoRa came from, which would be aided by the unlicensed spectrum modulating scheme's large connection budget.

To do this, the Lora infrastructure provides an improved modulation approach in combination with a multichannel multimodem transponder in the core network to process a large volume of information from many channels. Furthermore, by applying a distinct spreading factor to each signal, the broadcasting enables orthogonal segregation between them. This strategy has an advantage in terms of data rate management. For example, in the LoRa transmission technology [36], the connection between the needed data, chirp rate, symbol rate, and bit rate is as follows:

The bit rate of LoRa modulation *R*_*b*_,

(1) $$\begin{array}{c} R_{b}=SF*\frac{1}{\left[2^{SF}/BW\right]}\;\frac{bits}{\sec}\text{.} \end{array}$$

The spread factor is abbreviated as SF, while the modulation bandwidth is abbreviated as BW (Hz). According to equation (1), the spread factor SF and the bit rate *R*_*b*_ are equivalent and thus each ground station.

Discussing several areas of LoRa technology, such as LoRa deployments, LoRa application requirements, LoRa installation costs, LoRa emulators, and LoRa problems, is the survey's main contribution. According to the authors' knowledge, none of these LoRa technology-related problems have been addressed in a single study.

## 2. Background of LoRa

The IoT is an increasingly sophisticated generation of wireless connectivity, and LPWAN is the core infrastructure behind it. The patented technology LoRa [37, 38], created by SemTech in 2012, is perhaps the most advanced LPWAN. LoRa entails two layers, (a) a physical layer that makes use of CSS modulation [39] and a procedure for the MAC layer (LoRaWAN). It makes use of CSS. The CSS modulation system evolved from the DSS modulation method. CSS approaches allow one bit to be delivered for each chirp, requiring more bandwidth for data transfer than typical. A limited-band output is extended over a larger band with this approach, which makes it less effective regarding spectral usage but enhances the connection cost and improves immunity to interference, Doppler impact, and multipath fading. The network's gadgets employ orthogonal sequences or numerous channels, which boosts the overall platform efficiency. The LoRa technology allows several bandwidths of 125, 250, or 500 kHz [40]. The chirp signals' frequency varies over time at the transmitter, but the stage between the two end-to-end symbols remains constant. Suppose the chirp signals' frequency variations are gradual. In that case, more energy may be deposited per chirp representation, thus allowing the message to be decoded by the recipient at an attenuation level of 19.5 dB below the noise signal [41]. The emphasis on energy competence, coverage, and scalability distinguishes the LPWA technology [42]. Typically, these machinery activate in the unlicensed sub-1 GHz Industrial, Scientific, and Medical (ISM) band, which is a common band for all “Short Range” equipment [43] as low-interference gadgets (e.g., bells, identifying devices, broadcasting, metering, RFID, mobility solutions, alerting, and detecting). This frequency is also used by protocols like 802.15.4 g, Z-Wave, 802.11 ah, and 802.15.4 [44, 45].

**Figure 1:**
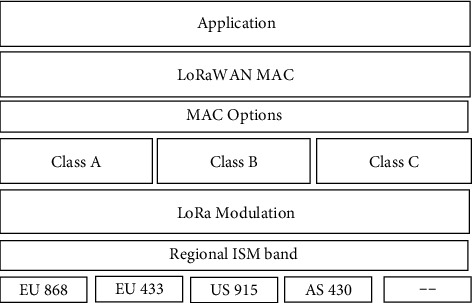
LoRaWAN and LoRA protocol.

The LoRaWAN protocol, established by the LoRa Alliance, governs the LoRa technology [46]. LoRaWAN is intended for end devices that are powered by batteries. In a star-of-stars architecture, a gateway often referred to as a collector or entry point node connects end systems to the primary networking controller [47]. End devices use single-hop LoRa or FSK transmission to connect to one or more gateways, whereas gateways and network servers use ordinary Internet protocol (IP) connectivity [48]. Both LoRa and LoRaWAN are seen in Figure 1. The end devices utilizing unlicensed radio spectrum resources in the ISM band use various channels and data rates [49]. According to research, the quality of service (QoS) is affected by sub-band selection and combination [16]. While maximizing Internet bandwidth and terminal battery lifetime, a dynamic data flow approach allows for individual terminal data rates and frequency [50]. The end devices choose channels in a pseudorandom method for each broadcast to reduce interference. They also ensure the maximal transmission duration and duty cycle are appropriate for the sub-band to use and comply with local laws [51].

LoRaWAN, comparable to any other ISM frequency standard, must abide by the rules set out according to the locale where the network is set up [52]. Beyond that, the LoRa network operator can specify the channels over which devices can interact, which can be in any of the subbands accessible. Multiple signals can be sent on the same channel concurrently since each possible LPWA usage scenario, and the spread factors are at minimum orthogonal sufficient [53]. LoRaWAN also establishes additional rules, such as prohibiting the usage of a subband for the following time-on-air^*∗*^(1/DutyCycleSubband1) seconds after a message is sent. This prevents a node from broadcasting a burst of messages on a certain sub-band until the duty cycle limit is achieved [54]. The gateway must also meet this time of sub-band requirement, reinforcing the need to reduce downlink traffic [55]. Simple gadget interaction is provided by Class A, a more complex gadget interaction is provided by Class B, and the gadget is given receiving windows at predefined intervals by Class C, which is identical to Class A but runs in constant reception status when it is not broadcasting [56].

The device may then connect by employing a basic ALOHA-based approach on the gateways once settings and channels are selected [57]. Because a larger spread factor equates to a higher number of chirps utilized for each symbol, higher spread factors result in greater energy consumption for each packet, demonstrating that the everyday output and equipment lifetime is inversely correlated with the range between the gadget and adjacent access point [58].  Table 2 illustrates the time identical packets with different spread factors were broadcast. However, regional laws' limiting access requirements, extra LoRaWAN regulations, and the shortcomings of the straight ALOHA-based medium access management, which is inappropriate for crowded and congested infrastructures [59], all limit the performance of a LoRaWAN network.

According to reference [60], LoRaWAN operates in an unlicensed frequency range to prevent interference and get the optimal spectrum characteristics. Furthermore, the frequency of operations varies from nation to country.  Table 3 depicts the frequency with which the LoRa technology is used worldwide. The radio planner is the tool from where users can check the frequency according to their use (https://www.wireless-planning.com/radioplanner).

### 2.1. Parameters of the LoRa Technology

To customize connection efficiency and energy usage, a LoRa module could be set using varied Transmit Power, Bandwidth, Carrier Frequency, Coding Rate, and Spreading Factor (SF). All of these are communication parameters of LoRa.

#### 2.1.1. Transmit Power

A LoRa radio transmit power could be increased in 1 dB increments from 4 dBm to 20 dBm. However, the span is frequently confined to 2 dBm to 20 dBm due to the device implementing limitations. Furthermore, energy values greater than 17 dBm might always be utilized with a 1% duty cycle due to physical constraints [61].

#### 2.1.2. Spread Factor

The symbol rate to chip ratio is known as the spread factor. The greater spreading ratio improves the power of a signal, and hence, the responsiveness and range of the signal; however, it usually extends the packet's duration. 2 SF is used to estimate the sum of chips per symbol. With 12 spread factors, for instance, 4,096 symbols were utilized. Each increase in spread factor reduces the propagation speed by half, doubling the transmission period, and as a result, power usage. The spreading factor might range from 6 to 12. As established in research, wireless transmissions with multiple spread factors are orthogonal to one another, and network segregation utilizing distinct SF is achievable [62].

#### 2.1.3. Coding Rate

The forward error correction rate adopted by the LoRa transmitter to prevent the surge of disturbance is termed coding rate, but this might be changed between 4/5, 4/6, 4/7, and 4/8. A greater coding rate provides better defense; however, it usually lengthens the time spent in the air [63]. The coding rate of the data is recorded in the preamble of the payload, which is usually decoded at a coding rate of 4/8. Therefore, transmitters with different coding rates can still interact with everyone else if they utilize an unambiguous preamble.

#### 2.1.4. Carrier Frequency

The primary frequency is known as the carrier frequency, which could be configured in 61 Hz levels within 137 and 1020 MHz, depending on the LoRa chip, this frequency may be limited to 860 MHz to 1020 MHz [64].

#### 2.1.5. Bandwidth

The length of a communication channel is referred to as a bandwidth or throughput. Greater bandwidth results in a better connection speed but lesser sensitivity (due to noise mixing). With a reduced bandwidth, the sensibility is better; however, the transmission flow is less. Decreased bandwidth necessitates greater precise crystals. A chip speed equivalent to the channel capacity is used to convey the data; for example, a bandwidth of 500 kHz is equivalent to a chip speed of 500 kbps. However, the bandwidth could be between 7.8 and 500 kHz. Most LoRa devices work at 500, 250, or 125 kHz [65].

## 3. Related Work

In this section, the author discusses a few recent survey papers that are pertinent to the study.

In this study [66], operations on LoRa-based platforms are examined. Their behavior is described, and they are categorized based on their technical advancements. Several performance factors are mentioned throughout this study that stands out. These variables fall into five major groups, which include the characteristics of the physical layer, installation and equipment characteristics, end device transmission parameters, LoRa MAC standards, and application requirements.

In this work [67], using the RSSI as a comparative metric, researchers provide a summary of the research conducted on LoRa signal disruptions. To compare them with the data gathered on the innovative framework of the Smart Village of Cozzano, a scientific initiative aiming to create electronic equipment for the monitoring and preservation of the environment, second, we extract the primary influences. The technical difficulties of installing the LoRa networks and current solutions are covered in-depth in this article's assessment of the LoRa networks [68].

Some of the unresolved problems of LoRa connectivity are presented based on our thorough research of existing solutions. This survey report aims to stimulate additional research on enhancing the LoRa network performance and allowing more useful deployments. In this paradigm, communities may test circular economy ideas for resource optimization, reducing waste, reuse, and recycling. A smart city model may benefit from data created by the elements of an Internet of Things (IoT) environment in two important ways: (1) by generating a circular economy and (2) by producing intelligence to help people and municipal officials make better decisions. In this environment, it is to our best advantage to comprehend the key IoT research axes, especially those built on the LoRa platform. Because LoRa is an open standard and helps to create sustainable smart cities, it has drawn the attention of academics [28].

This study [69] offers a method for classifying research articles with various subjects. The most current developments in LoRa research and use cases are also revealed by this study. Finally, this study will offer suggestions on how to effectively utilize the benefits of LoRa-based technologies in the creation of IoT systems and solutions for researchers and practitioners.

Researchers examine several LoRa application domains and associated activities in this article [70], as well as prospective chances for improvement and related factors. In addition, for improved performance, we provide a generic design to incorporate Edge computation capabilities in IoT-based applications.

This article [71] discusses LoRa's benefits over other IoT technologies already in use. It also talks about the characteristics of LoRa. The open-grade secure LoRaWAN (Long Range Wide Area Network) standard is used for IoT connection. The LoRa association is a free, nonprofit organization that collaborates to advance LoRa.

The LoRa technology is an area that is covered in all of the survey articles here. Every article's author discusses LoRa from the perspective of their study, as explained in this section. The LoRa technology is being explored in this study as well from a variety of perspectives, including deployments, application requirements, deployment costs, simulators, and challenges that are not addressed in the other studies mentioned above together.

## 4. Lora Deployments

Various research studies on low-power wireless technology have been conducted. This is expected to consider the experimental architecture of the LoRa/LoRaWAN standard and the availability of hardware for experimental study and rapid development. LoRa transmission has acquired popularity due to, in recent years, the scholarly society and industry have worked together.  Table 4 illustrates the deployment of LoRa applications in different areas like smart agriculture, smart metering, environment monitoring, and appliance monitoring, researchers in reference [72] make a LoRa-based large-scale agriculture farm monitoring system and examine the remote monitoring of farms concerning the flexibility of hardware, software, and platform [73]. The authors deploy the LoRa-based smart metering infrastructure and calculate the cost impact and energy use. Scholars [74] implement the LoRa-based atmospheric monitoring system and examine the system's performance using performance metrics like end-to-end throughput delay. Researchers in the article [75] deploy the LoRa-based smart appliance monitoring and controlling system and analyze the power usage of the system. The application of Lora in plants was proved in the research [76]. In reference [77], the IoT-monitored architecture relying on the Lora standard was described for the remote management of offshore sea fields [78, 81]. Furthermore, demonstrate that LoRa could be utilized for smart watering. Using LoRa connectivity for monitoring systems in the field, research in references [79, 80] followed an analogous strategy.

## 5. Coverage of LoRa for IoT

Various measures, including transmitted signal intensity level, signal-to-noise ratio, and packet transit rate, can be used to assess LoRa's range. SNR is a measure of connection quality to the atmospheric characteristics of a wireless network [82]. Depending on the sort of technology, the position of the devices, and the style of connectivity, such as point-to-point or gateway interfaces, The IoT devices in smart cities need varying service regions. Those factors are mostly related to the platform's design needs. For example, healthcare solutions consider completely fixed interactions between a screening framework and a central node based on our performance evaluation. However, a few apps, like many concentrating on new patient follow-up, need signal efficiency throughout communication whenever the devices travel [83]. The management of smart street lighting equipment in traffic management or mobility applications necessitates connectivity between several terminals positioned inside a limited range. Approximately 35 meters [84] is the ideal spacing between roadway lights. Despite using a smart device [85], the RSSI level may be utilized to establish the user's location in smart parking. If the RSSI number is high, the person is close to a parking spot.

**Figure 2:**
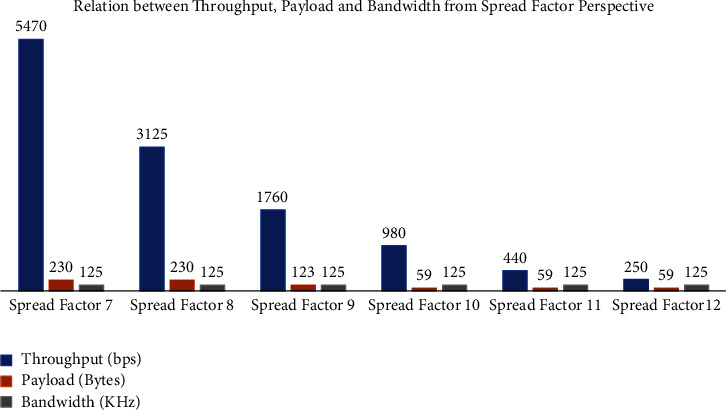
Relationship among throughput, payload, and bandwidth with spread factor.

In the LoRa technology, Figure 2 depicts the relationship between throughput, payload, and bandwidth. When the spread factor is set to a high value, the throughput and payload are reduced. For example, if the specified Spread Factor is 7, the throughput is 5470 bps, and the payload is 230 bytes for the same bandwidth of 125 kHz at a band of 868 MHz. When the spread factor is set to 12, the throughput drops to 250 bps, and the payload shrinks to 59 bytes.

## 6. Requirements of Low Power Applications

The large variety of low-power wireless deployments leads to a considerable diversity within the parameters. Gadgets can provide ranges from extremely short to long areas, from stable to movable locations, reduced power battery links to corporate electricity links, and favorable to dangerous environments [86]. Some deployments, such as interior fixed installations, have a relatively confined coverage spread. Comprehensive communication availability is required for gadget movement scenarios, like resource tracking [10]. Compared to crucial IoT services that demand exceptionally low latency and exceptionally high dependability, the vast IoT application sector necessitates connections for a large range of gadgets. A substantial portion of low-power large area systems rarely deliver short messages, are delay tolerant, do not demand significant data throughput, and have minimal power usage and cost requirements [87]. The features can always be transformed into a list of criteria. The primary needs are M2M traffic processing, huge throughput, energy saving, reduced power processes, expanded range, privacy, and interconnections [88].

### 6.1. Longer Battery Lifetime

Despite user contact, the IoT gadgets are predicted to get an extended lifetime. As a result, mesh layouts were used in various industries, significantly shorter platforms. Mesh architecture, on the other hand, is connected in addition to massive implementation costs. Furthermore, the necessity of mesh connections to traverse data to numerous nodes might result in certain nodes burning the entire battery, rapidly, lowering network lifespan [89]. Therefore, it is advised that low-power wireless network solutions use the star network architecture to achieve low power usage while providing long distance communications or a vast covering region [90].

### 6.2. Range

LPWAN must usually offer long-distance connectivity back closer to 10–40 kilometers in rural and 1–5 kilometers in urban zones, with *a *+* *20 decibels increase over traditional mobile infrastructures [6]. Interior coverage and difficult-to-reach regions such as subterranean sites and tunnels might be required for data management and collection apps. In addition, the coverage should meet the requirements for adaptive connection speeds and regulated information error ratios [91]. Although the shorter frequencies of the sub-GHz range have superior distribution properties than the 2.4 GHz and above ranges, most LPWANs can accomplish durable and consistent connection with reduced power expenditure [92]. Because of the increased penetration, network structure installation ought to be countrywide, necessitating the simplicity of integrating equipment, and smart objects. In concept, this indicates that methods must conform to particular specifications, in particular, to maximize the accessibility of intelligent objects and allow smooth networked communication [93].

### 6.3. Requirements for Traffic

The fundamental connectivity process of low-power wireless networks includes data produced by dispersed devices. However, despite the data generated by cellphones or other gadgets, low-power wireless data can differ in various aspects, such as the number of messages, message size, and dependability needs. This is because low-power wireless technologies have various applications with various specifications. For example, some applications are delay tolerant, but others, like flame monitoring, nuclear radioactivity detection, and house protection need priorities and fast delivery [94].

### 6.4. Technological Complexity

The development of small sized minimal complex gadgets is becoming a key necessity in managing a large number of gadgets, low budget maintenance, and extended area penetration [95]. The decreased equipment complexity design reduces energy usage in battery powered gadgets while losing efficiency. In summary, the gadgets are intended to have limited computing power. Furthermore, equipment must support simple communication design and standards [96]. In terms of technology, wireless transmitters must be versatile and software reconfigurable to accomplish the requisite flexibility of low power wireless equipment [97].

### 6.5. Pervasive Connectivity

One of its fundamental features is the ability to access the Internet of Things from everywhere all the time [87]. Becoming widespread is another aspect, although connecting a large volume of gadgets is important, particularly considering that the volume of IoT-connected gadgets surpasses mobile network subscribers [98]. Furthermore, it is anticipated that the concentration of the linked equipment will not be constant, necessitating the ability to communicate a large volume of gadgets at once. The infrastructure could support multicultural gadgets. Numerous gadgets might interact with one another and other networks and technologies by utilizing similar spectrum resources, which would lower service efficiency [99]. Therefore, low-power wireless gadgets should be able to communicate with and function in various low-power wireless platform contexts while having the capacity to tolerate, handle, and mitigate interruptions. Regardless of the equipment design or application platform, the network must be capable of allowing peripheral communication [42]. Various network solutions must provide smooth end-to-end coexistence. Interoperability and adaptable bridges across different connectivity systems could be necessary for this. We anticipate complete end-to-end platform compatibility [100].

### 6.6. Privacy and Security

Due to their enormous number, weaknesses, and ease of use, low-power wireless equipment has very strict security criteria [101]. Assistance must be provided for the fundamental characteristics of identification, verification, confidence, secrecy, data protection, and nonrepudiation. Assaults, including harmful programs (like viruses), hackers using low-power wireless equipment and systems, managing wiretapping, and sniffer threats, including denial of service, should all be dealt with by the security staff [102]. Protecting the equipment's identification and position from prying eyes is also crucial. Furthermore, as needed for diverse purposes, this should enable safety for both onward and reverse communication [103]. Therefore, any infrastructure must meet the two concerns of confidentiality and protection [104]. This is essential to ensure that the low-power wireless networks created will support a wide range of connected applications [105]. Every connection in the network will be guaranteed to be legitimate by the LPWAN-based protection. The application level can incorporate additional security features [106], but it is outside the scope of this research.

## 7. Cost of Deployment

The low-power wireless approaches are primarily a trade-off between cost, available bandwidth, and installation expense [107]. Financial restrictions are frequently cited as impediments to the widespread acceptance of innovation. Equipment and network installations should be kept to a minimum, and the cost of the object must be kept to a minimum as well, with neither GSM chip required. Moreover, the infrastructure should be simple and require little management. Complications impact expenses, which is why Intelligent things' software and hardware must be simple [108]. Gadgets and operating costs are essential in low-power wireless scenarios. In addition to the platform's conventional criteria for cheap implementation and running costs, the enormous volume of gadgets engaged places significant limits on the expense, operating expenditures, and the need for minimal energy usage. The ability to upgrade software without replacing equipment is a critical feature that must be offered. Furthermore, it is critical to provide flexibility, ease of implementation, and service [109].

## 8. LoRa Simulators

**Figure 3:**
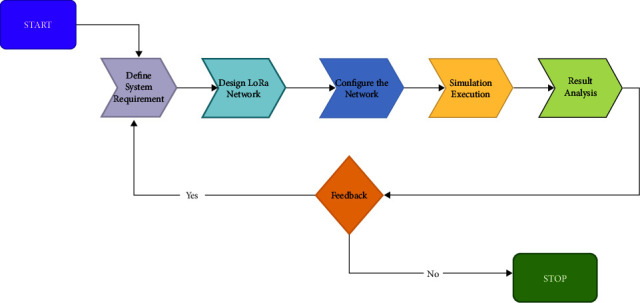
A diagram showing how the simulation process works.

Network architecture with particular specifications is a difficult process considering that the criteria ought to be adequate for the specific circumstance or application, not unique. As a result, it is critical to employ a simulator. Simulation and prototyping on computers are the best ways to deepen your understanding of a system's performance and assess strategies for its operation in innovative or predictive ways. A strategy that considers simulating is known as computational methods, physical and mathematical terminology, and engineering formulae to characterize a system's behavior and performance in intangible world case studies. Generally, a simulator developer should give the user the ability to choose the networking architecture, each station's main characteristics, the connections between them, the potential traffic pattern, and any traffic routing methods. The LoRa network simulator is more important since it can be used to build and assess a LoRa-based application without requiring costly installation before the framework is executed. The area of LoRa provides a wide range of high-end, open-source modeling packages [110]. These many LoRa numerical simulations were developed and used to test various LoRa situations. The simulation process is shown in Figure 3.

To correctly define the demands and environment of the system, users must initially adequately articulate the challenge to be addressed, as illustrated in Figure 2. The next step might be to simulate this structure throughout to define the crucial factors and fine-tune the details. Then, launch the experiment and observe the results come next. Eventually, after examining such data, users may either go through each step again employing the instructions the simulation program gave them, or they can stop the process and review the results. The scientific community has access to numerous simulation environments, including PhySimulator, FLoRa, and LoRaSim. Below these simulators are explained in more detail. The LoRa simulators are shown in this section.

### 8.1. FLoRa

The OMNeT++ continuous episode simulation toolkit, on which the FLoRa simulator is based, is widely used in continuous event simulations and freely available for nonprofit and academic usage, according to the Academia Creative Commons. Regardless of being built on the OMNeT++ framework, the INET Approach is also used by FLoRa. It is an unlicensed repository for OMNeT++ that aids in the experimenting process for various network protocols. C++ is used to create FLoRa [111]. To test the performance of the LoRa network using the ADR mechanism, build a LoRa (FLoRa) simulator's architecture. It demonstrated the ADR's effectiveness in enhancing the PDR while improving energy efficiency [112]. The OMNeT++ library, which is an open-source, was created to aid in the testing of various network protocols. C++ is used to write FLoRa code, which allows for the creation of LoRa networks to support the incorporation of LoRa stations, controllers, and server modules [113]. Separate modules attached to a network server can be used to construct application logic. Dynamic configuration settings are supported by the network server and nodes, which are managed by the ADR and consider the acquisition effect and collisions. The module features precise backhaul network modeling and the ability to simulate numerous gateways. Energy consumption information in each node and throughout the whole network may be obtained at the end of the simulation [114]. The built simulator package includes a sample case inside the FLoRa experiment directory. The scenario includes many elements that imitate a network with ten nodes distributed arbitrarily in square system architecture and one gateway connected to a network server. According to an exponent with a defined mean, each node sends one packet at a time. Several parameters must be chosen while simulating a LoRa network, including simulation duration, warm-up period, SF, signal strength for every LoRa end device, backhaul network architecture, and connections. Following the experiment's conclusion, the scenario metrics and trace files are created. As shown in Figure 3, simulation statistics may be seen using the OMNeT++ GUI [114].

### 8.2. LoRaSIM

SimPy is used to implement the LoRaSim, a custom-built discrete-event simulator. SimPy procedures are described by Python generator methods and may be used to represent active components such as consumers, automobiles, and agents, among other things. SimPy also has many shared resources that may represent congested areas with limited capacity (like servers, checkout counters, and tunnels) [115]. In addition, it enables two-dimensional gridding of the LoRa stations. The LoRaSim is developed in Python version 2.7. It also uses the Python packages SimPy, NumPy, and Matplotlib. This program includes four separate simulations, each of which focuses on different network and node attributes. There are models for single base stations and simulations for up to four base stations. The sort of antennae used in the simulations may also be classified since there are files that model terminals with directional antennas. You may alter many parameters in this simulator, including the number of nodes and base stations to emulate.

Furthermore, you may select a comprehensive and simplified collision, and check, the number of the LoRa networks to be checked, the simulation period, and the range between two base stations. When LoRaSIM is run, if the visuals parameter is assigned to 1, it displays some charts. Still, most of the data is provided via the command-line interface because it ultimately outputs to an expX.dat file. There is no graphical interface except the plot representations, and the user must operate through the command line. Because the initial version of LoRaSim only supported the uplink, many improvements were suggested to make it versatile and assist the downstream. As a result, it may assess efficiency factors like electricity use and scalability. Many data points are demonstrated on the cmd prompt and imported in Name.dat, which can be examined with various graphical programmers.

### 8.3. PhySimulator

The LoRa connection scenario is simulated by a basic emulator called PHY Simulator. PhySimulator is developed in the coding MATLAB. This simulator's objective is to assess the receipt of multiple LoRa transmissions that overlap and interfere with different spreading factors. The packet, symbol, and bit error rates are displayed eight figures after each program execution. The results are the bit error rate, symbols, and packets for each spreading factor, as well as the total bit error rates, symbols, and packets for all other spreading factors [116]. The user may alter numerous settings like program parameters' values being modified using this simulator. You could, for instance, change the payload bits, bandwidth, and maximum attempts per step. Unfortunately, the client must explicitly change each of these settings in the MATLAB program as there is no visual tool available to edit them.  Table 5 shows a comparison of the simulators used in the LoRa technology.

Understanding which framework to utilize and which development tools to learn for the particular issue is crucial for the user and whether or not it is open source, especially for the scientific community and academics. This is required to expand current tools to include new scientific features, innovations, and as with the module, the capacity to use “infrastructure” for various standards, such as LoRa and already-existing procedures. We look into if there are any associated books, websites, or communities that support each piece of software using this framework. Furthermore, having some graphical data and statistics is vital to comprehend and analyze the simulation results to validate your system model. Thus, this aspect is also investigated.  Table 5 shows a comparison of the characteristics of each simulation program. As seen in Table 5, all of the characteristics of the examination simulators are compared line by line, and we can conclude that all simulators accessible are pretty competent.

To begin with, all three simulators are discrete-event simulators. This suggests that they represent the environment as a time-domain series of discrete occurrences. This enables the simulator to go on to the next event without watching the system continuously, given that the system does not change between two consecutive events. In summary, FLoRa is written in C++, PhySimulator is written in MATLAB, LoRaSim is written in Python, and the LoRaWAN module is written in C++ and Python. In contrast to other simulators, FLoRa through OMNeT++ offers PhySimulator, LoRaSim, MATLAB, and PyChrome environments in that sequence, whereas just a few plots do. According to each simulator's official website, FLoRa and PhySimulator have more relevant publications. However, PhySimulator has been upgraded to another version called LoRa+. According to the Scopus database, LoRaSim has several related papers.

In comparison to PhySimulator and FLoRa, the LoRaSim modules have more publications. As a result, several simulators include additional information on their websites about the installation procedure and how to utilize the equipment. They are all free software, and GitHub is where you can get their program code.

## 9. Classification of Problems in LoRa

Researchers look at the difficulties of LoRa communication. Understanding how these issues affect the functionality of the LoRa system reveals how serious they are. Following are some challenges identified during the study.

### 9.1. Resource Allocation

The primary purpose of the resource control strategy is to align the reception window of the access points with the transmitting windows of edge devices. Understanding that the base station transmitting windows do not always remain open is important because they are used to signal when data has been received [117]. In addition, end devices cannot constantly have their transmitting and reception windows open because of constraints on the energy economy. As a result, this discrepancy should be resolved while considering network density, limiting such techniques' effect to increase LoRA capacity. As a result, numerous end devices might transmit simultaneously and concurrently thanks to the clever management of logical and frequency channels. When applying clever resource assignment strategies, encouraging results were seen in references [118, 119].

### 9.2. Communicative Distance

A crucial LoRa architecture foundational feature is a broad transmission distance. LoRa system uses a chirp spread spectrum that is better resistant to interruption. In a variety of settings, including residences, hospitals, universities, and forests, LoRa connectivity could be used. Terminal sites will include those exposed to air, enclosed by metal or brick. Signal attenuation, transmission loss, and fading must be combated with precisely various installation circumstances in mind to increase information penetrating, and subsequently, LoRa network availability [120]. The ability of gateways to receive signals under a particular limit but unable to understand them has been reported. A method must be developed to decipher such signals to increase the transmission distance. The estimation of LoRa system availability is a significant additional difficulty.

### 9.3. Large Volume of Gadgets

Mobile communications may inspire scholars to develop novel densifying solutions for LoRa infrastructure in light of its limitations, like the duty cycle and limited capacity [121]. This is a time-tested response to the growing connection requirement in a dense metropolitan setting such as the metropolitan hub [122]. In addition, the ability of LoRa to link a greater number of terminal devices may be considerably increased by combining the access points densified with the method.

### 9.4. Energy Usage

The considerable energy consumption of LoRaWAN is its main crucial feature. This represents a crucial factor in extending the life of edge gadgets. LoRa bridges are anticipated to function for a prolonged time of 5 to 10 years using little servicing [123]. Therefore, one of the biggest problems with LoRa connectivity is power usage. In saving energy, the LoRa system uses two strategies: (1) Immediate bandwidth uses for the chirp signal and (2) avoiding the need for complex MAC methods for planning. Despite these methods, peripherals still utilize higher energy than anticipated due to unforeseen events, such as packet forwarding brought on by fading channels [124].

### 9.5. Communication Security

Security is a key problem in digital connectivity in general [125]. Snooping, opportunistic sending and station cloning are only a few examples of vulnerability threats [126, 127]. The keys utilized for cryptography are the targets of all assaults listed above. The system as a whole may be destroyed if this password is hacked. Currently, the LoRa system utilizes symmetrical secret cryptography using AES-128-bit encoding; however, the code is created and never updated using current LoRa hardware [128, 129]. Therefore, the procedure for key creation and updating is a critical problem. Furthermore, third-party clearance is necessary whenever the connection providers provide, and platform providers are separate. Therefore, many apps need third-party clearance to protect user protection and confidentiality [111, 130].

## 10. Challenges of LoRa

### 10.1. Scalability

LoRa installations will link many endpoint gadgets engaged in detecting activities. In addition, to comprehend the scalability of this innovation, it is vital to evaluate its capability. Therefore, studies on LoRaWAN's capability for individual port installations and urban deployments have been conducted. According to the analysis of transmission speeds and propagation distance, it is recommended that urban-sized LoRa implementations employ the greatest bit rate. In fact, given a connection speed slower than the maximum one, the terminal occupancy per area remains unchanged under the assumption provided.

### 10.2. Interference

LoRa communication is comparable to a pure ALOHA scheme because it would not specify any specific spectrum admission management. However, one of the drawbacks of ALOHA-based solutions is their blind broadcasting method, which enables the broadcaster to broadcast anytime there is a packet to transmit requiring channel monitoring. Furthermore, in an ALOHA-only system, the susceptible period is double the cycle duration. This implies that every duplicate broadcast that begins in the timeframe interval that opens one signal period earlier and closes after the signal's broadcast will destroy the transmission.

### 10.3. Security

Considering the explosive development of LoRa connections and the assumption that LoRa has several weaknesses, privacy, and security are crucial. However, Lora's physical characteristics have revealed new and potent assaults which are difficult to defend against, and the significant power efficiency demand also makes it difficult to implement effective defense. Furthermore, whereas physical layer security techniques can potentially guarantee complete protection, their use is restricted by their lack of robustness. For instance, the current key creation techniques often let just two valid parties arise throughout a lengthy querying time, although never multiple parties.

## 11. Conclusion

The low-power wireless technology LoRa has gained significant research traction throughout the previous several years, stimulating a wide range of studies. This article provides a detailed analysis of the LoRa networks and a quick rundown of the technology's specifications. The researchers explained the history of LoRa deployments, issues encountered while setting up LoRa networks and current solutions. In this study, the discussion also included LoRa simulation tools. Some unresolved challenges of LoRa communication are also discussed based on our thorough research of existing solutions. This study aims to stimulate additional research on enhancing LoRa network performance and allowing more useful deployments.

## Figures and Tables

**Table 1:** Characteristics of LoRA technology [21].

Characteristics	LoRA
Range	10 km (approximately)
Modulation	Chirp spread spectrum
Data rate	<50 Mbps
Frequency	Europe (433, 868 MHz), United States (915 MHz), Asia (430 MHz)
Power usage	Low

**Table 2:** LoRA spread factor.

Spread factors	Packets	Tx (ms)	Bandwidth (kHz)	Chirps per symbol
7	417	71.936	125	128
8	224	133.632	125	256
9	121	246.784	125	512
10	66	452.608	125	1024
11	30	987.136	125	2048
12	16	1810.432	125	4096

**Table 3:** Frequencies of LoRA in various regions.

Regions	Frequency bands (MHz)
European Union	433
North America	902–928
Australia	915–928
China	470–510
Japan	920–925
Korea	920–925
India	865–867
Europe	863–870

**Table 4:** Deployments of LoRa applications.

References	Year	LoRa deployments	Assessments
[72]	2022	Monitoring of large-scale agriculture farms	Examine remote monitoring with flexibility
[73]	2021	Smart metering	Cost and energy analysis
[74]	2021	Ecological monitoring in infrastructure-less areas	Analysis of performance metrics
[75]	2021	Monitoring of appliances	Examine power usage
[76]	2020	Crop monitoring	Analyse power consumption
[77]	2020	Monitoring of sea farm	Assessment of scenarios
[78]	2020	Water management	Examination of experimental performance
[79]	2020	Monitoring of crops and plants	Analyse the environmental impact
[80]	2020	Monitoring the crop	Assessment of energy usage
[81]	2019	Irrigation management	Valuation of experimental results

**Table 5:** Comparison of LoRa simulators.

Simulator features	FLoRa	LoRaSIM	PhySimulator
Operating system	macOS, linux, windows	macOS, linux, windows	macOS, windows
GUI	Yes	Only plot	Only plot
License type	Source is open	Source is open	Free
Installation requirements	OMNeT++ 6 and INET 4.3.1	SimPy, NumPy, matplotlib	MATLAB
Language type	C++	Python	MATLAB
Support	Limited	Limited	Good
Popularity	Medium	High	High

## Data Availability

No data were used to support this study.
